# Population genetic patterns among social groups of the endangered Central American spider monkey (*Ateles geoffroyi*) in a human-dominated landscape

**DOI:** 10.1002/ece3.547

**Published:** 2013-04-12

**Authors:** Suzanne Hagell, Amy V Whipple, Carol L Chambers

**Affiliations:** 1 School of Forestry, Northern Arizona UniversityFlagstaff, Arizona, 86011; 2 Merriam Powell Center for Environmental Research and Department of Biological Sciences, Northern Arizona UniversityFlagstaff, Arizona, 86011

**Keywords:** *Ateles geoffroyi*, conservation, human-dominated landscape, inbreeding, microsatellites, spatial genetic structure

## Abstract

Spider monkeys (Genus: *Ateles*) are a widespread Neotropical primate with a highly plastic socioecological strategy. However, the Central American species, *Ateles geoffroyi*, was recently re-listed as endangered due to the accelerated loss of forest across the subcontinent. There is inconsistent evidence that spider monkey populations could persist when actively protected, but their long-term viability in unprotected, human-dominated landscapes is not known. We analyzed noninvasive genetic samples from 185 individuals in 14 putative social groups on the Rivas Isthmus in southwestern Nicaragua. We found evidence of weak but significant genetic structure in the mitochondrial control region and in eight nuclear microsatellite loci plus negative spatial autocorrelation in Fst and kinship. The overall pattern suggests strong localized mating and at least historical female-biased dispersal, as is expected for this species. Heterozygosity was significantly lower than expected under random mating and lower than that found in other spider monkey populations, possibly reflecting a recent decline in genetic diversity and a threat from inbreeding. We conclude that despite a long history of human disturbance on this landscape, spider monkeys were until recently successful at maintaining gene flow. We consider the recent decline to be further indication of accelerated anthropogenic disturbance, but also of an opportunity to conserve native biodiversity. Spider monkeys are one of many wildlife species in Central America that is threatened by land cover change, and an apt example of how landscape-scale conservation planning could be used to ensure long-term persistence.

## Introduction

**Figure 1:**
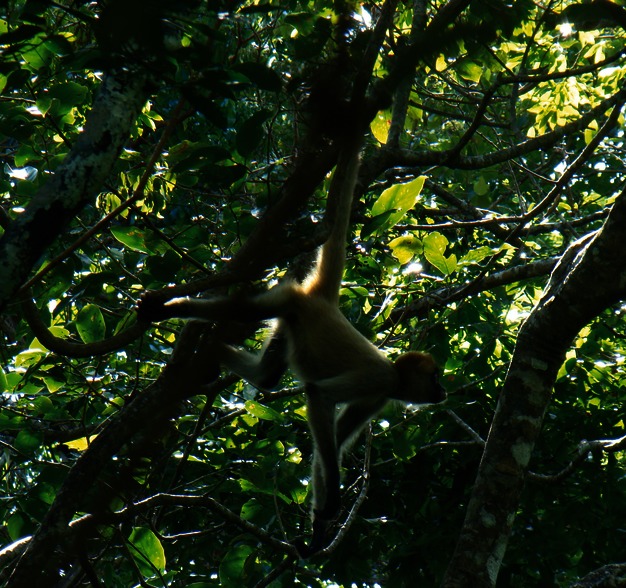
*Ateles geoffroyi* on the Rivas Isthmus, Nicaragua. Photo credit: Bill Noble. This photo was taken on 8 January 2010 (UTM 16P 637627E 1241983N).

The black-handed spider monkey, *Ateles geoffroyi* (Fig. 1), like its genus as a whole, is a primate that is widespread in its distribution but locally threatened. Historically, *A. geoffroyi* spanned all wet and dry forests less than 3000 m in elevation from southern Mexico through Panama and across both coasts (Ford 2006; Rylands et al. 2006). Until recently, the species was considered to be of low conservation concern because of this wide distribution, but was re-listed in 2008 as endangered due to extensive deforestation across Central America (IUCN 2010). The other widespread sympatric primates, *Alouatta palliata* and *Cebus capucinus* are not considered to be declining at the same rate and are of Least Concern (IUCN 2010).

Despite a highly adaptable ecological strategy, spider monkeys are likely to be sensitive to anthropogenic threats. Spider monkeys have a unique, highly plastic ecological strategy among Neotropical primates in that the size and composition of the social group, daily foraging parties, and territorial ranges are adjusted based on the distribution of resources (Di Fiore et al. 2008). The diet of spider monkeys also appears adaptable to regional and seasonal food availability (Russo et al. 2005; Suarez 2006; Di Fiore et al. 2008; González-Zamora et al. 2009). However, spider monkeys are also arboreal, and as such are reliant on the closed-canopy forests at risk from loss and fragmentation. They are also popular targets for hunting and the pet trade (in Central America, Peres 2001; Duarte-Quiroga and Estrada 2003; Estrada 2006), and as such directly impacted by an increased density of humans.

The resiliency of spider monkey populations to these threats is further undermined by their slow reproductive rate. Ateline primates have the “slowest” life history of all primates other than the great apes (oldest age at first reproduction and long interbirth interval for body size; Di Fiore et al. 2011) and the lowest intrinsic rates of population increase among Neotropical mammals (Robinson and Redford 1994). Spider monkey females reach reproductive maturity only after 5 years of age and interbirth intervals exceed 2–4 years (Ramos-Fernández and Wallace 2008; Di Fiore et al. 2011). Atelines are also rare among primates for having female-biased dispersal (e.g., Di Fiore et al. 2009) which could be additionally detrimental to population stability if females face a higher risk of mortality when dispersing.

The complex interaction of flexibility and sensitivity makes it difficult to predict under what conditions spider monkeys can persist in Central America and what management strategies will be effective to conserve them. Because of their close relationship with forests, it is commonly assumed that spider monkeys are intolerant of forest loss, which suggests that the biogeography of the genus could have also been historically shaped by forest cover (Collins and Dubach 2000; Collins 2008). However, recent observations of Central American spider monkeys suggest a complex and highly variable response to forest fragmentation and land cover change. While decline and extinction have been documented in many protected areas, this is matched by observations of long-term persistence in disturbed agricultural landscapes (Estrada 2006; Estrada et al. 2006; Ortiz-Martinez et al. 2008). Protection from hunting combined with forest restoration in Costa Rica has resulted in accelerated population growth at Corcovado National Park (Weghorst 2007), but only resulted in slow recovery at Guanacaste National Park (Sorensen and Fedigan 2000). To further complicate our understanding of spider monkey resilience, Muñóz et al. (2008) uncovered a relict population of spider monkeys in central Chiapas, Mexico that use steep canyon walls to access distant forest patches; forests in this area would have otherwise appeared to be insufficient to maintain a population.

In this study, we analyzed neutral genetic diversity across a human-dominated landscape in Nicaragua to better understand the viability of spider monkey populations in disturbed landscapes. Collins and Dubach (2000) posited that the slow rate of population turnover in spider monkeys could have prevented the divergence of some subspecies during temporary forest refugia events. Thus, spider monkeys may be unlikely to differentiate when isolated over relatively short periods of time. Instead, spider monkeys may be able to maintain genetic diversity when isolated into smaller populations, so a lack of genetic structure and high diversity in our study population would suggest that this species could persist in disturbed landscapes (Ford 2006). However, considering the high rate of habitat loss and elevated conservation status, we took the more conservative hypothesis that the long history of agriculture and forest disturbance on this landscape (e.g., predating Spanish conquest in 1527; Staten 2010) has resulted in restricted migration and subsequent differentiation among demes on the landscape accompanied by a lower genetic diversity compared to spider monkeys in continuous forest.

## Materials and Methods

### Study area and sample collection

**Figure 2:**
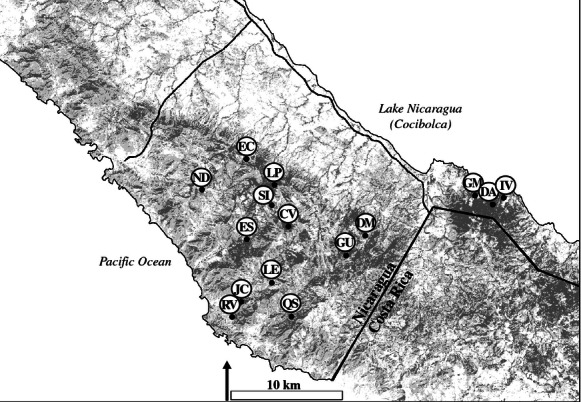
Distribution of the 15 sampling sites or putative social groups of spider monkeys (*Ateles geoffroyi)* on the Rivas Isthmus, Nicaragua used in this analysis. Site locations reflect the centroid of a 3 ha sampling area. Dark gray shading indicates closed-canopy forest cover, light gray is open-canopy, and lines represent permanent roads and the international border (forest cover derived from Sesnie et al. 2008).

The Rivas Isthmus is a narrow land corridor connecting the Pacific slope of Central America (Fig. 2). As in all of the dry forests in Mesoamerica, the Rivas Isthmus has been populated for centuries and consequently has a long history of anthropogenic disturbance. It is estimated that less than 0.1% of the original old growth, Mesoamerican dry forest remains (Janzen 1988; Gillespie et al. 2000), and this forest type is currently a high priority for conservation (Miles et al. 2006). However, Rivas retains significant cover of closed canopy dry forest and therefore has the potential to support a diverse animal and plant community.

We surveyed for spider monkeys across a 50,000 ha area on the isthmus, bordered on all sides by actual or potential barriers to animal migration. The Costa Rican border forms the southern boundary and the northern boundary is a major two-lane road. Both features are buffered by deforested land and nonnative, planted forests that do not provide habitat. Spider monkeys may have been extirpated north of this study area possibly because it is more heavily populated and urbanized. Spider monkeys are found in forest patches south of the study area, along the southern border of Lake Nicaragua. Reconnecting these patches could enable gene flow between spider monkeys on the Pacific and Atlantic coasts.

We stratified sample collection based on expected social group structure as spider monkeys are not continuously or randomly distributed across the landscape. They likely have localized patterns of genetic similarity that result from the size and distribution of social groups, female-biased dispersal, and the species' preference for closed canopy forest. As such, we searched for animals or fresh samples within 300 ha plots, or within the expected home range size for a social group (95–390 ha; Wallace 2008; Spehar et al. 2009). Most sampling locations were chosen based on the presence of large, closed-canopy forest stands (coverage described in Sesnie et al. 2008). Informal interviews with local residents and behavioral observations were also used to confirm the likelihood that samples came from a single social group. Spider monkeys fission into small foraging parties and actively avoid human contact, which makes locating animals difficult. Hence, we used a concentrated searching technique (Pruetz and Leasor 2002) to maximize the number of genotypes potentially represented in the fecal samples, focusing on riparian areas and large, fruit-bearing trees. We collected fecal samples within 8 h of defecation and stored them in a 1:1 solution with RNALater for 1–3 months in ambient temperatures prior to DNA extraction or long-term storage at −20°C. Most samples were collected during the dry season (January–May).

We collected fecal samples from 2007 to 2010 to describe genetic variation within each social group. We retained groups for population-level analysis that included >10 samples; observed group sizes in *Ateles* spp. are 15-55, in *A. geoffroyi* 18-42 (Wallace 2008). This resulted in separate global and restricted datasets for both of the genomes reported below. Not all sites were sampled in all years, and sampling was suspended at any site that was impacted by significant land cover change. For example, forest was cleared on several private properties near Sapoá that likely resulted in a change in the home range of spider monkeys at two sites (DM and GU; Fig. 2). Hence, these sites were sampled between 2007 and 2008, before these changes took place. Because of the slow reproductive rate in this species, it is unlikely that we sampled multiple generations; in dry forests, females do not reproduce until approximately 7 years of age, and interbirth intervals can exceed 48 months (Fedigan and Rose 1995).

### DNA extraction and species identification

We extracted DNA from fecal samples using QIAmp DNA Stool Mini Kits with an extended proteinase K digestion step, to maximize DNA yield. We confirmed the source species of all mammalian DNA by analyzing two overlapping, 400–500 bp segments of the mitochondrial cytochrome-b region using recommended protocols (Janczewski et al. 1995; Verma and Singh 2003). It is necessary to confirm the species identity for all fecal samples as fecal morphology can be misleading, particularly if there are sympatric animals that have a similar diet. In our study, this procedure removed morphologically similar samples of primates (*A. palliata* and *C. capucinus*) and kinkajou (*Potos flavus*). The polymerase chain reaction (PCR) products were purified using ExoSap-It (USB) and sequenced using the standard BigDye Terminator 3.1 (Applied Biosystems, Foster City, CA) or stepped elongation cycle sequencing protocols (Platt et al. 2007). Sequencing reactions were purified using the recommended ethanol precipitation method and analyzed on an ABI 3730 Genetic Analyzer. All samples that matched the *Ateles* genus in the GenBank database were retained for analysis; that is those with identity scores of 100% and e-value >0 using nucleotide BLAST within Geneious Pro 5 (Biomatters, Auckland, New Zealand).

### Analyses of the mtDNA control-region

We used information from Collins and Dubach (2000) and Ascunce et al. (2003) to develop primers within the first hypervariable portion of the mitochondrial control region (HVRI). Our forward (5′ GTGCATTATTGCTTGTCCCC) and reverse (5′ GTTGGTTTCACGGAGGATGG) primers are similar to those in Ascunce et al. (2003), but with minor sequence shifts to better match spider monkey template DNA, minimize the risk of hairpinning, and increase GC content in the 5′ end. This primer pair results in a 221 bp amplicon that overlaps with those produced by Collins and Dubach (2000). PCR reactions were optimized in 20 μL reactions at the following concentrations: 0.5 units of Taq polymerase, 1× PCR buffer, 1.5 mmol/L MgCl_2_, 0.2 mmol/L dNTP mix, 0.5 μg BSA, and 0.1μmol/L of each primer with 4 μL of the extracted DNA solution. We used touchdown PCR profiles for the HVR1 and microsatellite markers (described below) to ensure amplification. Annealing temperatures ranged from 68 to 51°C with starting and denaturation at 94°C and extension at 72°C. The profile was as follows: 94°C (5 min), 17 cycles [94°C (30 sec)], 68-51°C (30 sec each, −2°C/cycle), 72°C (30 sec)], 23 cycles [94°C (30 sec)], 50°C (30 sec each), 72°C (30 sec)], and 70°C (5 min). Sequencing protocols follow those used for species identification above. The resulting sequences were manually edited, aligned, and checked against accessioned sequences in Geneious Pro 5. Samples with identical mtDNA haplotypes that also displayed identical multilocus nuclear microsatellite genotypes (described below) were considered to be duplicate samples of the same individual and were removed. We calculated diversity statistics and tests of neutrality and variance in DNASP (Librado and Rozas 2009) and Arlequin 3.5.1 (Excoffier and Lischer 2010) and used these to construct a haplotype network in HapStar (Teacher and Griffiths 2011). We further tested the significance of differentiation among sampling sites using on Hudson et al. (1992) method for high diversity datasets (for all tests *P* < 0.05, 10,000 permutations).

### Analyses of nuclear microsatellite genotypes

We screened 23 nuclear microsatellite primer pairs previously amplified in platyrrhine primate species and chose eight easily scored and polymorphic loci to avoid downstream genotyping errors (DeWoody et al. 2006; Supporting Information). We tested both M13 universal tail (Schuelke 2000) and directly labeled primers. PCR was prepared in 10 μL volumes with 3 μL of template, and a final concentration of 0.3 units of taq polymerase, 1× PCR buffer, 2 mmol/L MgCl_2_, 0.2 mmol/L dNTP mix, 0.5 μg BSA, and 0.02 μmol/L of each primer. Some sample DNA was more successfully amplified by increasing the MgCl_2_/dNTP ratio. We used touchdown PCR around the optimal annealing temperatures to ensure amplification of all alleles: 95°C (5 min), 35 cycles [95°C (45 sec)], Ta (45 sec), 72°C (45 sec)], and 72°C (7 min). All alleles were analyzed on an ABI 3730 and were scored in GeneMapper 4.0 (Applied Biosystems).

We took several precautions to avoid genotyping errors, which is a particular concern when using noninvasive samples to amplify nuclear DNA (e.g., DeWoody et al. 2006). A blood sample from a captive *A. geoffroyi* (Hogle Zoo, Utah) and human buccal swabs were genotyped for all loci as positive controls, to predict allele size, and to assess contamination. Alleles were discarded if they resembled those found in human DNA rather than the spider monkey control. Two independent observers scored alleles visually (without automated binning) and we replicated PCR reactions to confirm our results. Following replication trials, samples that were missing data for more than two of the eight loci were removed from the analysis. We checked for indications of null alleles, allelic dropout, and stuttering based on patterns of homozygosity and allele size using MicroChecker (van Oosterhout et al. 2004). We matched multi-locus genotypes, reviewing the chromatograms for all genotypes that differed by fewer than three alleles. We used this final dataset to estimate the probability of identity across loci and genotypes, using both the unbiased estimator and the conservative P(ID)sib (in Gimlet; Valière 2002; Waits et al. 2001).

Population-level statistics were calculated in Genepop 4.0 (Rousset 2008), SMOGD (Crawford 2010), and SPAGeDi (Hardy and Vekemans 2002), unless otherwise noted. We tested for linkage disequilibrium in the eight loci using the log-likelihood *G*-test. We assessed allelic and genotypic diversity via allele and private allele richness (rarefaction calculations based on one minus the minimum number of alleles at a locus in Hp-rare 1.0; Kalinowski 2005), expected and observed heterozygosity with Levene's correction for small samples sizes, and diversity and differentiation statistics (Hs, Nei's Gst, and Jost's D as estimated in SMOGD; 1000 bootstrap). Whereas Nei's “coefficient of differentiation” (Gst) may represent the effect of migration rates, Jost's D may better illustrate differentiation based on mutation rate and allele identity (Jost 2008, 2009). To determine if the observed level of heterozygosity was significantly lower than expected, we tested for homoscedasticity (Bartlett test) and conducted a paired *t*-test for deviations greater than zero (*P* < 0.05; in the R package adegenet 1.2-6; Jombart 2008). To identify deviations from Hardy Weinberg Equilibrium (HWE) we performed Raymond and Rousset's (1995) multisample test for heterozygote deficiency and Fisher exact tests with MCMC sampling (100 batches, 1000 iterations per batch; Guo and Thompson 1992).

To describe population structure and differentiation, we calculated Fst using the standard method of moments estimation (Weir and Cockerham 1984) and Jost's D. Although Hedrick's G'st as a standardization of Nei's Gst is a robust metric for highly variable markers or when comparing across markers, it does not correct for bias due to small sample or population size (Meirmans 2006). Furthermore, heterozygosity-based statistics may be biased if both diversity and population structuring are high, as both affect the partitioning of variance. Jost's D specifically incorporates effective alleles and genetic identity, information which is lost when using heterozygosity alone (Jost 2008). We tested for isolation by distance (IBD) in the population using Mantel tests in the vegan R package (Oksanen et al. 2005). We used two genetic distances among sites, the linearized Fst (to standardize Fst under IBD; Rousset 1997) and differentiation (D; Jost 2008). Spatial distances were calculated as Euclidean distances from a central location in each study site (measured in ArcGIS 9.3; ESRI, Redlands, CA), and were log-transformed for analysis (Rousset 1997).

To further investigate localized mating patterns, we compared relatedness coefficients between individuals among and within sampling sites and geographic distance classes (Hardy and Vekemans 2002). We chose Loiselle et al.'s (1995) kinship coefficient as it is independent of HWE and robust to localized, discontinuous mating patterns, and low frequency alleles (Vekemans and Hardy 2004). Kinship was calculated among individuals in predefined subpopulations (i.e., the putative social groups based on sampling site) and among 10 distance classes, ranging from 2.5 to 25 km (the longest distance between sites). Distance classes were designed to include an equal frequency of pairwise comparisons (Aspi et al. 2009).We used our entire sample of individuals (not just those within groups of >10 individuals) to analyze distance classes, in order to use all available information. SPAGeDI's jackknife procedure was used to summarize over loci and estimate standard errors. We ran 10,000 permutations of individual spatial locations for all analyses (Hardy and Vekemans 2002).

## Results

### Diversity in the mitochondrial control region

**Figure 3:**
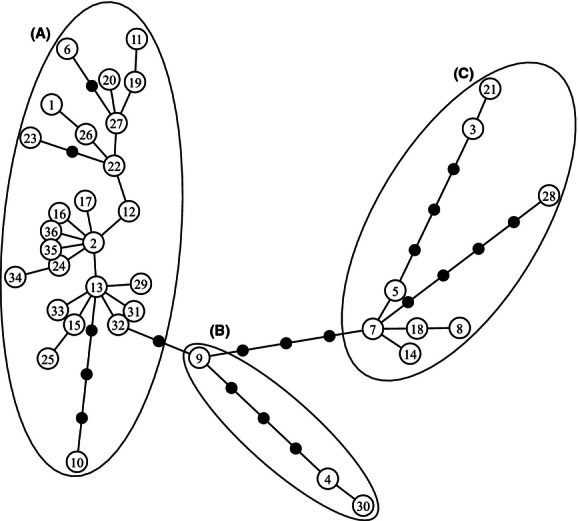
Haplotype network for the restricted dataset of 36 mitochondrial control region haplotypes from 162 spider monkeys (*Ateles geoffroyi*) on the Rivas Isthmus, Nicaragua.

We amplified the control region sequence from 185 individuals across 15 study sites and found 39 haplotypes. Nine study sites contained at least 10 unique individuals, our cutoff for inclusion in population-level analyses (Table 1). Hence the restricted dataset included 162 individuals, 36 haplotypes, and 31 polymorphic sites (including one indel). Diversity and theta(s) values among the sampling sites were variable. Overall haplotype diversity was 0.88 and 19 haplotypes were found at only one site. All haplotypes are similar and there are few mutational steps within the haplotype network (Fig. 3). Using this network, we defined three haplogroups based on the pattern of reciprocal monophyly. Haplogroup “A” encompassed 75% of the individuals and the two additional haplogroups are also found at most sampling sites (Table 1). The mean value of Tajima's D was negative, reflecting an excess of low frequency polymorphisms, but the test for non neutrality was nonsignificant (TD = −0.68; *P* > 0.05). Finally, differentiation among sampling sites was weak but significant (within-subpopulation variance = 86.41%, among-subpopulation variance = 13.59%, Hudson's test *P* < 0.05).

**Table 1:** Genetic diversity in the mitochondrial control region (HVR1) of spider monkeys (*Ateles geoffroyi*) in sampling sites from the Rivas Isthmus, Nicaragua in the restricted (*n* > 10) and global datasets (all *n*)

	*n*	*H*	*h*	*π*	*θ*s	SD	Haplogroup
Restricted (*N* = 162)							
IV	32	13	0.88	0.01	3.97	1.52	A,B,C
GM	28	11	0.92	0.02	3.85	1.52	A,B,C
ES	18	8	0.88	0.01	2.91	1.32	A,B
GU	17	9	0.90	0.02	5.03	2.09	A,C
ND	16	8	0.88	0.01	2.71	1.27	A,B
DM	14	6	0.87	0.02	4.40	1.20	A,B,C
LL	14	7	0.87	0.02	3.77	1.71	A,B,C
QS	13	5	0.81	0.02	4.51	2.02	A,B,C
DA	10	6	0.84	0.01	1.76	1.01	A,B
Global (*N* = 185)							
EC	8	4	0.75	0.02	4.63	2.23	–
JC	6	5	0.93	0.02	4.38	2.39	–
SI	6	6	1	0.03	6.13	3.21	–
CV	1	–	–	–	–	–	–
LP	1	–	–	–	–	–	–
RV	1	–	–	–	–	–	–

*N*, dataset sample size; *n*, sampling site sample size; *H*, # haplotypes; *h*, haplotype diversity; *π*, nucleotide diversity; *θ*s, Watterson's theta; Haplogroups shown in Figure 3.

### Microsatellite-based population diversity and structure

We collected microsatellite-based genotypes of 163 unique individuals from 13 sampling sites, and retained information for a restricted sample of 119 genotypes from six sites. The probability of identity for these individuals is below the recommended threshold of 0.001 (1.140e-08 for unrelated and 1.003e-03 for related individuals). There was no consistent pattern of gametic disequilibrium; any pair of loci that were significantly related at (*P* < 0.05) were only so at three or fewer study sites. Amplification results for all loci and individuals are described in Supporting Information. The Leon02, LL1110, and SB38 primers were adjusted to avoid PCR errors and florescent dye quenching. We found that the universal M13 tail system (Schuelke 2000) for labeling primers provided a prohibitively low yield of product, probably due to excessive nonspecific amplification. As a result, final genotyping was conducted with only directly labeled primers. Two tetranucleotide loci (Ab12 and Ce121) also showed evidence of interruptions in their repeat structure.

Genetic diversity as measured by allelic richness and expected heterozygosity was high in almost all loci and lowest in the tetranucleotide loci, Ce121 and AB12 (Table 2), which are expected to have a lower mutation rate (Ellegren 2000). Observed heterozygosity across loci also differed significantly from expected (*P* = 0.004, 95% CI = 0.117; Bartlett *P* = 0.9). All loci except for AB12 deviated strongly from HWE and tested positive for heterozygote deficiency across the restricted and global datasets. Inbreeding coefficients (Fit and Fis) were >0 in all loci (mean Fis = 0.14, Fit = 0.18).

**Table 2:** Genetic diversity in eight microsatellite loci from spider monkeys (*Ateles geoffroyi*) from the Rivas Isthmus, Nicaragua in the restricted dataset (*N* = 119)

Locus	*n*	Allele size range	*A*	Ho	He	Gst	*D*	HWE (*P*)	*U* (*P*)
D8S260	118	212–236	15	0.8	0.86	0.02	0.15	0.00	0.03
Leon02	114	186–198	8	0.46	0.72	0.06	0.17	0.00	0.00
SB38	118	137–153	9	0.72	0.84	0.01	0.07	0.00	0.00
LL1110	111	203–219	9	0.69	0.76	0.02	0.08	0.00	0.00
LL1118	113	134–148	9	0.44	0.71	0.06	0.15	0.00	0.00
LL157	111	217–235	10	0.68	0.77	0.01	0.05	0.00	0.00
Ce121	118	193–221	10	0.58	0.64	0.02	0.04	0.02	0.01
AB12	112	193–213	8	0.33	0.36	0.02	0.01	0.78	0.01

*n*, individual genotypes; *A*, allelic richness; Ho/He, observed/expected heterozygosity; Gst, Nei's relative differentiation (estimated); *D*, Jost's actual differentiation (estimated); HWE, exact test; *U*, test for heterozygote deficiency.

Genetic diversity across the six study sites that included >10 individuals was moderate (He = 0.63–0.74; Table 3), all sites exhibited low allele and private allele richness as estimated by rarefaction, and Fis values summarized across loci were variable but moderate to high at some sites. Mean kinship values were low within sites and overall (mean jackknifed estimation across the global dataset = 0.05), but observed similarity was significantly higher than expected (*P* < 0.05). There were six dyads that represent potential parent-offspring or sibling pairs (*k* > 0.5) within sampling sites, or IV (3), GU (2), and GM (1). Hence, samples from these sites may contain more than one generation.

**Table 3:** Genetic diversity summarized across eight nuclear microsatellite loci of spider monkeys (*Ateles geoffroyi*) in sampling sites from the Rivas Isthmus, Nicaragua in the restricted (*n* > 10) and global datasets (all *n*)

	*n*	*A*	HWE (*P*)	*U* (*P*)	Ar	Apr	Ho	He	Fis	*k*
Restricted (*N* = 119)										
IV	38	63	0.00	0.00	5.58	0.32	0.56	0.70	0.23	0.03
GM	24	53	0.00	0.00	5.50	0.17	0.64	0.70	0.12	0.02
GU	17	44	0.08	0.35	4.74	0.15	0.63	0.63	0.06	0.06
DM	15	52	0.00	0.00	5.78	0.35	0.62	0.74	0.20	0.02
ES	15	48	0.00	0.00	5.25	0.25	0.42	0.63	0.39	0.08
DA	10	48	0.22	0.06	5.90	0.53	0.70	0.72	0.02	0
Global (*N* = 162)										
LL	9	37	0.01	0.00	–	–	–	–	–	–
ND	8	32	0.00	0.00	–	–	–	–	–	–
JC	7	32	0.18	0.08	–	–	–	–	–	–
QS	7	34	0.00	0.00	–	–	–	–	–	–
SI	4	26	–	–	–	–	–	–	–	–
LP	1	15	–	–	–	–	–	–	–	–

*A*, allelic richness; HWE, exact test; *U*, test for heterozygote deficiency; Ar, allelic richness (rarefaction); Apr, private allele richness (rarefaction); Ho/He, observed/expected mean heterozygosity; *k*, mean kinship jackknifed over loci.

Pairwise Fst values between sampling sites were low, even between pairs separated by long spatial distances (Table 4). However, permutation tests revealed that the Fst values were significant in most pairwise comparisons. Furthermore, estimates of pairwise differentiation based on allele identity (Dst) closely mapped the expected values in that they were highest among spatially distant sites. Both Fst and Dst matrices had a strong and significant correlation to Euclidean distance between sites (Fst|Euclidean distance: *r* = 0.63, *P* = 0.01; D|Euclidean distance: *r* = 0.55, *P* = 0.04). As expected in populations with low heterozygosity, D and Gst provided similar results in all locus and population comparisons hence only D is reported.

**Table 4:** Pairwise Fst and differentiation (Jost's D) of six sampling sites of spider monkeys (*Ateles geoffroyi)* from the Rivas Isthmus, Nicaragua in the restricted dataset (*N* = 119)

	GM	GU	DA	DM	ES	IV
GM	–	0.085	−0.059**	0.189	1.37	0.85
GU	0.07*	–	0.213	0.712	1.15	1.04
DA	0.01	0.04*	–	0.127	1.17	0.03
DM	0.01	0.07*	0.02	–	1.07	1.10
ES	0.05*	0.13*	0.05*	0.02	–	1.33
IV	0.06*	0.08*	0.00	0.05*	0.09*	–

Lower, Fst; Upper, Jost's D;

* *P* < 0.05 based on 1000 permutations,

** For Dest across loci [from SMOGD], negative values should be considered as zero.

**Figure 4:**
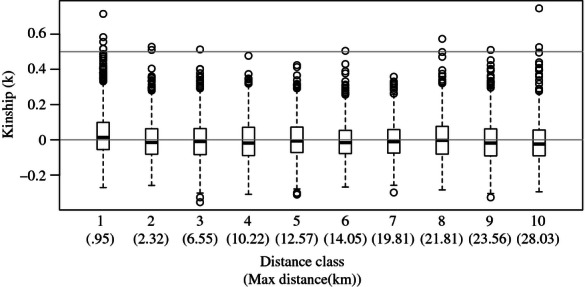
Pairwise kinship (Loiselle et al. 1995) between 162 spider monkeys (*Ateles geoffroyi*) across 10 distance classes on the Rivas Isthmus, Nicaragua. Max Distances report the upper limit of distances within each distance class. Line intercept at *k* = 0 is equivalent to a null probability of identity by descent, *k* > 0.5 indicates 1st order relatives.

When analyzing spatial autocorrelation in kinship across the global sample of 163 individuals, and without assigning subpopulations based on sampling site, the regression of kinship to spatial distance was negative and significant (*b* = −7.34E-07, *P* = 0.001). The smallest distance class (individuals ≤ 1 km apart) maintained the only mean kinship value above 0.005 (range among all 11 distances classes = −0.01 to 0.03; Fig. 4) and demonstrated a significantly higher observed kinship compared to expected (*P* > 0.05). The largest distance classes (approximately 22–28 km) exhibited the lowest mean kinship and significantly lower observed kinship than expected, despite having dyads with high kinship values (*k* > 0.05; Fig. 4) and one dyad with the highest estimated kinship for the dataset (*k* = 0.75).

## Discussion

### Genetic structure and diversity

High haplotype diversity, haplotype similarity, and weak population structuring in the mitochondrial control region suggests that this spider monkey population was at least historically panmictic and experienced female dispersal. The deviation from neutrality due to an excess of low-frequency polymorphisms could also indicate that the population is recovering from a bottleneck event. However, rapidly mutating nuclear microsatellites are more likely to reflect recent population patterns than mitochondrial DNA, particularly as ateline primates have exceptionally slow reproductive rates. Mitochondrial gene regions may only be useful for longer time frames; Collins and Dubach (2000) used the same gene region to resolve specific and sub-specific relationships in the spider monkey genus. However, these results are consistent with expectations, as ateline primates are expected to have either bisexual or female-biased dispersal (Di Fiore et al. 2009).

Our suite of nuclear microsatellites indicate spatial genetic structuring due to geographic distance, local mating, and close-kin associations within social groups. IBD explained a high proportion of the variance in differentiation among sampling sites and this was corroborated when we looked more closely at fine-scale patterns of kinship across all sampled individuals. We specifically found spatial autocorrelation over small distances and a significant reduction in close kin relationships over distances greater than 25 km. These results confirm the effect of localized mating on gene flow, as individuals within sampling sites (i.e., within 3 km^2^) are similar by descent relative to random expectation. This also suggests a limitation on dispersal distances and an increased risk from inbreeding and declines in genetic diversity due to drift. One dyad in the largest distance class exhibited a high estimated kinship, possibly demonstrating a sibling pair that is spatially distant. We cannot confirm if this represents a long-distance dispersal event or an artificial translocation event (e.g., captive release).

### Loss of genetic diversity

We expected a clear indication of the effects of forest cover loss on genetic structure via differentiation; instead these data suggest intense localized mating that could lead to a decline in overall genetic diversity. The comparatively weaker pattern of genetic structure in the maternally-inherited mitochondrial marker reinforces the theory of female-biased gene flow. However, most nuclear loci and sampling sites also had significantly lower than expected heterozygosity, consistent with a general loss of diversity. The diversity found in these loci is also lower than that reported for other spider monkey populations. Di Fiore et al. (2009) analyzed six of the same microsatellite loci to describe spider monkey (*Ateles belzebuth*) social groups in relatively intact forests in Yasuní National Park, Ecuador. Their results showed markedly more genetic diversity than in our sample. For example, the Yasuní social groups exhibit approximately the same number of alleles within individual social groups as was found in our entire population and higher expected heterozygosity (Yasuni sites He = 0.79–0.86; Rivas sites He = 0.63–0.74).

There are multiple explanations for heterozygote deficiency due to locus and population-specific phenomena (Allendorf and Luikart 2007). The three most common explanations are the presence of null alleles (locus-specific), discontinuities in gene flow as individual demes reach fixation in different alleles (the Wahlund Effect on genetic drift; subpopulation-specific), or inbreeding and population decline (neither locus or subpopulation specific). In this study, the deficiency in heterozygotes was not locus or subpopulation specific, suggesting that inbreeding or population decline is likely. We do not consider null alleles to be at issue in this study as all loci violate HWE or have low diversity, most sampling sites are out of HWE, and five of these loci that were also studied in the Yasuní spider monkey population did not exhibit heterozygote deficiency (Di Fiore et al. 2009). It is unlikely that null alleles would persist in multiple loci and multiple primer sets per loci.

An alternative explanation is that our pooled samples contain more than one deme. Fredsted et al. (2005) found a similar pattern of high homozygosity and low but significant pairwise Fst among solitary gray mouse lemur (*Microcebus murinus*) groups and concluded that the predefined groups encompassed more than one reproductive subdivision. However, in our sample, estimations of allele and private allele richness are comparable across the study sites when sample size is taken into account.

### Conservation of Ateles in Central America

This study suggests that there is a genetic consequence for forest-dependent spider monkey populations from the accelerated human pressures on forest systems in southwestern Nicaragua. Collins and Dubach (2000) suggested that spider monkey populations were not likely to diverge genetically over short evolutionary time periods because of their slow reproductive rate. Our results from nuclear microsatellites suggested that over short periods, isolation combined with population declines leads to a loss of genetic diversity and increase differentiation among social groups. Human disturbance of this landscape has occurred for over 400 years, but the current pattern of population decline is most likely due to the recent escalation of these pressures, including habitat loss and overharvesting for hunting or pet trade.

The heightened risk of inbreeding due to localized mating within social groups of spider monkeys is of substantial conservation concern. We found evidence of reduced diversity in nuclear DNA across almost all loci and sampling sites. This strongly suggests that functional diversity is also at risk in these animals. In primates, inbreeding depression has been linked to a variety of congenital malformations in wild populations and explicitly responsible for disease susceptibility in captivity (Charpentier et al. 2007, 2008). Inbreeding may result in high neonatal and infant mortality and ultimately lowered overall recruitment (Charpentier et al. 2008). Indeed anecdotal evidence and recent survey data support this phenomenon in the Rivas population of spider monkeys, where we regularly observe infants in all seasons but not juveniles (K. Williams-Guillen, S. Hagell, S. Otterstrom, S. Spehar, C. Gomez, pers. obs.). Information from non neutral genes may help determine if the reduction in neutral diversity seen here is consistent with inbreeding depression (e.g., the Major Histocompatibility Complex (MHC); Knapp 2005; Radwan et al. 2010).

Restoring and maintaining connectivity across landscapes could enable the species to adapt to ongoing change. If these populations are losing genetic diversity, then maintaining gene flow is even more important. Connectivity with other spider monkey populations, such as those that are southeast of our research area, would greatly enhance the future viability of the Rivas population. However, threats to spider monkeys are not limited to the current availability of genetic diversity, but the continued conversion of forest habitat and dispersal corridors as well as hunting and animal capture. Given the precarious status of this population and the long recovery period that will likely be needed to restore genetic diversity, this landscape will require the protection from further animal extraction, conservation of core areas for habitat, connections between cores for migration, in addition to support for gene migration from outside populations.

## Supporting Information

**Data S1.** FASTA alignment of the mitochondrial control region, global dataset (*n* = 185).

**Data S2.** Data file with information on samples used in the analyses.

**Table S1.** Further details on the microsatellite loci screened for use in this study, including any edits to the primer sequences and references.
