# Supplementary material for: Population genetic patterns among social groups of the endangered Central American spider monkey (Ateles geoffroyi) in a human-dominated landscape
Source: Ecol Evol. 2013 Apr 12;3(5):1388–99. doi: 10.1002/ece3.547 (PMC3678491; doi:10.1002/ece3.547)
Supplement: Supplementary file 1 [file ece30003-1388-SD1.docx]

**Supporting Information**

Table A. Primer sequences and amplification results from 23 nuclear microsatellite loci analyzed using noninvasive genetic samples from *Ateles geoffroyi* (Rivas, Nicaragua) as described in Hagell et al. (2013).

| Locus | Exp Size (bp) | Obs Size (bp) | Exp Ta* (°C) | Obs Ta* (°C) | Edited** Primer | Final** Forward | Final** Reverse | Results | Additional Notes | Reference |
| --- | --- | --- | --- | --- | --- | --- | --- | --- | --- | --- |
| Ab12 | 233-285 | 203, 250 | 60, 65 | 70-60 | - | aaatcaaggcccacaggg | caaaggcaagaaagcaagaag | Polymorphic | - | Goncalves et al., 2004 |
| Ab20 | 242-304 | 220 | 67 | >60 | Forward | tgtggtggtgggtgcgc | ttgcttttccccttttgtgtttgc | Nonspecific | Guanine at 5’ | Goncalves et al., 2004 |
| AP6 | 190 | - | 47 | - | Reverse | agtgttttatggtttgagat | - | No Amplification | Published Reverse is incorrect | Ellsworth & Hoezler 1998 |
| AP68 | 240, 190 | 170 | 50 | 54-50 | - | tgttggtataatctttccta | acatacacctttgagtttct | Monomorphic | - | Ellsworth & Hoezler 1998; Cortes-Ortiz 2009 |
| AP74 | 145-160 | - | 52, 50 | - | - | tgcacctcatctctttctctg | catctttgttttcctcatagc | No Amplification | - | Ellsworth & Hoezler 1998; Cortes-Ortiz 2009 |
| Api09 | 462-470 | - | 60 | - | - | acttgctgtgtgaccttcag | aatgtctatccagcagcctct | No Amplification | - | Cortes-Ortiz et al., 2009 |
| Apm01 | 178-219 | - | 64 | - | - | cacgtgtgtccagcttgtct | attctgctgcccttgagttc | No Amplification | - | Cortes-Ortiz et al., 2009 |
| Apm09 | 174-186 | - | 55 | - | - | cagggttcctctttcactgg | ttgggatcacaagtgcttca | Nonspecific | - | Cortes-Ortiz et al., 2009 |
| Ceb121 | 140–184 | 193-221 | 59, 50 | 60-58 | - | ccatttaggggaggagaagg | ttggttggtaggcaggtagg | Polymorphic | - | Muniz & Vigilant 2004 |
| D17S804 | 144-169 | 146 | 60 | - | Both | cctgtgctgctgataacc | cactgtgatgagatgtcat | Nonspecific | Guanine at 5’, Self-anneal or hairpin | Cortes-Ortiz et al., 2009; DiFiore et al., 2009 |
| D5S111 | 162-191 | 188 | 58-48 | 55-58 | Forward | catcattttagaaggaaat | acatttgttcaggaccaaag | Low Amplification | Guanine at 5’ | Weber et al., 1990; Witte & Rogers 1999; Cortes-Ortiz et al., 2009 |
| D8S165 | 115-148 | - | 58-48 | - | - | acaagagcacatttagtcag | agcttcatttttccctctag | Nonspecific | - | Cortes-Ortiz et al. 2009 |
| D8S260 | 180-214 | 194-234 | 56-60 | 60 | - | aggcttgccagataaggttg | gctgaaggctgttctatgga | Polymorphic | - | Ellsworth & Hoezler 1998; DiFiore et al., 2009 |
| Ham01 | 150-204 | 170 | 57 | 60-50 | - | tcctcaagaatgtctacctg | ggtaatagatagatgacggatggg | Monomorphic | - | Katoh et al., 2009 |
| Ham11 | 133-175 | 153 | 57 | 49-46 | - | ctgggacattttgattgtcg | ttgggctagatcttggcatt | Poor Amplification | - | Katoh et al., 2009 |
| Leon15c85 | 270-280 | - | 60 | - | - | ctgatccttgaagcagcattg | ggttaaaggggttcgttctgtg | Duplicate chromosome | - | Perez-Sweeney et al., 2005 |
| Leon2 | 206-212 | 188-204 | 55 | 56-59 | Reverse | ctgcttcttgttccacttcttctc | gtttgggtggttgccaa | Polymorphic | Self-anneal or hairpin | Perez-Sweeney et al., 2005 |
| Leon21c75 | 274-282 | 250 | 60-62 | 59-62 | Reverse | cagttgagggaacaggaatta | actgcactgacagagcaag | Poor Amplification | Self-anneal or hairpin | Perez-Sweeney et al., 2005 |
| LL1110 | 202-222 | 203-221 | 53 | 50-55 | Forward | tgaatgagagaatcaaag | tatgttccacagtagaaagc | Polymorphic | Guanine at 5’ | DiFiore & Fleischer 2004; DiFiore et al., 2009 |
| LL1118 | 128-165 | 124-144 | 50 | 53-55 | - | tttctccctctcagattaccag | ccttgaggtttttgggttcc | Polymorphic | - | DiFiore & Fleischer 2004; DiFiore et al., 2009 |
| LL157 | 215-223 | 217-229 | 53 | 55 | - | tggcaagtctggtttcaagc | ttccagactgagctaggatgc | Polymorphic | - | DiFiore & Fleischer 2004; DiFiore et al., 2009 |
| LL312 | 186-195 | - | 50 | - | Forward | agacaacgacattaacaatgc | gcttctggtttctgattgagg | No Amplification | Guanine at 5’ | DiFiore & Fleischer 2004 |
| SB38 | 127-147 | 137-151 | 50 | 50-55 | Forward | cctcaatgggttttaacc | agaacgagtctgtatcttga | Polymorphic | Guanine at 5’ | Bohle & Zischler, 2002 |

Exp and Obs TA = expected and observed annealing temperatures, Edited and Final Primers = which (if any) primers were edited and the sequences used in this study.

References

Bohle UR, Zischler H (2002) Polymorphic microsatellite loci for the mustached tamarin (*Saguinus mystax*) and their cross-species amplification in other New World monkeys. Mol Ecol Notes 2:1-3.

Cortés-Ortiz L, Mondragón E, Cabotage J (2010) Isolation and characterization of microsatellite loci for the study of Mexican howler monkeys, their natural hybrids, and other Neotropical primates. Conserv Genet Resour 2:21-26.

DiFiore A, Fleischer R (2004) Microsatellite markers for woolly monkeys (*Lagothrix lagotricha*) and their amplification in other New World primates (Primates: Platyrrhini). Mol Ecol Notes 4:246-249.

DiFiore A, Link A, Schmitt C, Spehar SN (2009) Dispersal patterns in sympatric woolly and spider monkeys: integrating molecular and observational data. Behaviour 146:437-470.

Ellsworth JA, Hoezler GA (1998) Characterization of microsatellite loci in a New World primate, the mantled howler monkey (*Alouatta palliata*). Mol Ecol 7:657-666.

Goncalves E, Silva A, Barbosa M, Schneider M (2004) Isolation and characterization of microsatellite loci in Amazonian red-handed howlers, *Alouatta belzebul* (Primates, Plathyrrini). Mol Ecol Notes 4:406-408.

Katoh H, Takabayashi S, Itoh T (2009) Development of microsatellite DNA markers and their chromosome assignment in the common marmoset. Am J Primatol 71:912-918.

Muniz L, Vigilant L (2008) Isolation and characterization of microsatellite markers in the white-faced capuchin monkey (*Cebus capucinus*) and cross-species amplification in other New World monkeys. Mol Ecol Resour 8:402-405.

Perez-Sweeney B, Valladares-Padua C, Burrell A, Di Fiore A, Satkoski J, van Coeverden De Groot P, Boag P, Melnick D (2005) Dinucleotide microsatellite primers designed for a critically endangered primate, the black lion tamarin (*Leontopithecus chrysopygus*). Mol Ecol Notes 5:198-201.

Weber JL, Kwitek AE, May PE (1990) Dinucleotide repeat polymorphisms at the D5S107, D5S108, D5S111, D5S117 and D5S118 loci. Nucleic Acids Res 18:4035.

Witte SM, Rogers J (1999) Microsatellite polymorphisms in bolivian squirrel monkeys (*Saimiri boliviensis*). Am J Primatol 47:75-84.
